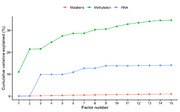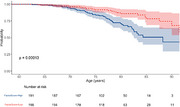# Multiomics Patterns and Alzheimer’s Disease Risk

**DOI:** 10.1002/alz70861_108392

**Published:** 2025-12-23

**Authors:** Michael Vacher, Tenielle Porter, Lidija Milicic, Eleanor K. O’Brien, Milcent Tsvangiray, Ameya S Kulkarni, Vinny Vijayakumar, Annita Martin, Erin Murphy, Elizabeth Asque, Claire Konefal, Sara Ishak, Keysury Curro, Magdalena Rogozinska, Fedik Rahimov, Jeffrey Waring, Aparna Vasanthakumar, Simon M. Laws, James D. Doecke, AIBL Research Group

**Affiliations:** ^1^ Edith Cowan University, Joondalup Australia; ^2^ Australian E‐Health Research Centre, CSIRO, Perth, Western Australia Australia; ^3^ Centre for Precision Health, Edith Cowan University, Joondalup, Western Australia Australia; ^4^ Centre for Precision Health, Perth, Western Australia Australia; ^5^ AbbVie Inc, North Chicago, IL USA; ^6^ AbbVie, Inc., North Chicago, IL USA; ^7^ School of Medical and Health Sciences, Edith Cowan University, Joondalup, Western Australia Australia; ^8^ School of Medical and Health Sciences, Edith Cowan University, Perth, Western Australia Australia; ^9^ The Australian e‐Health Research Centre, CSIRO, Brisbane, QLD Australia

## Abstract

**Background:**

Alzheimer’s disease (AD) is an increasing societal burden globally, with an urgent need for early screening strategies to improve disease management. Multiomics profiling represents a promising approach for identifying biomarkers and stratifying disease risk.

**Method:**

We analysed data from a cohort of 391 individuals (mean age: 73.6 ± 6.7 years) enrolled in the Australian Imaging, Biomarkers, and Lifestyle (AIBL) study. Multi‐Omics Factor Analysis (MOFA) was employed to integrate and identify key axes of variation (latent factors) across three omics datasets: RNA sequencing (N = 19259), single nucleotide polymorphisms (SNPs, N = 298516), and DNA methylation (N = 154846). Latent factors were subsequently assessed with PET Amyloid (Spearman’s Rho) and time to progression (accounting for age), using Cox proportional hazards models. Time to event was derived as time for a participant to progress from MCI to AD, or time the participant remained MCI (censored).

**Result:**

The unsupervised integration using MOFA showed that the principal axes of variation captured combined contributions from all three omics datasets. DNA methylation explained the largest proportion of variance, followed by RNA sequencing and SNP genotype data (31.8%, 13.9%, and 0.9% cumulative variance explained across the first 10 factors, respectively). Post hoc analyses showed that one of the factors was correlated with PET Amyloid levels (Rho = 0.20, controlling for age and APOE ε4). This factor also significantly predicted time to event, with participants exhibiting higher scores progressing to AD more quickly (Figure 2).

**Conclusion:**

This study demonstrates the utility of multiomics integration for uncovering biologically relevant patterns in AD and its potential application disease risk stratification. Future research should focus on validating these patterns in larger, independent cohorts and exploring their utility in clinical applications.